# The triglyceride to high-density lipoprotein cholesterol (TG/HDL-C) ratio as a predictor of insulin resistance but not of β cell function in a Chinese population with different glucose tolerance status

**DOI:** 10.1186/s12944-016-0270-z

**Published:** 2016-06-07

**Authors:** Meicen Zhou, Lixin Zhu, Xiangli Cui, Linbo Feng, Xuefeng Zhao, Shuli He, Fan Ping, Wei Li, Yuxiu Li

**Affiliations:** Department of Endocrinology, Key Laboratory of Endocrinology, Ministry of Health, Peking Union Medical College Hospital, Beijing, 100730 China; Nankou Community Health Service Centers, Changping District, Beijing, 102200 China; Nankou Railway Hospital, Changping District, Beijing, 102200 China; Department of Nutrition, Peking Union Medical College Hospital, Beijing, 100730 China

**Keywords:** Lipid ratios, Glucose tolerance status, Insulin resistance, β cell function

## Abstract

**Background:**

Triglyceride/high-density lipoprotein-cholesterol (TG/HDL-C) ratio was a surrogate marker of IR; however, the relationship of TG/HDL-C with IR might vary by ethnicity. This study aims to investigate whether lipid ratios-TG/HDL-C, cholesterol/high-density lipoprotein-cholesterol (TC/HDL-C) ratio, low-density lipoprotein-cholesterol/high-density lipoprotein-cholesterol (LDL-C/HDL-C)) could be potential clinical markers of insulin resistance (IR) and β cell function and further to explore the optimal cut-offs in a Chinese population with different levels of glucose tolerance.

**Methods:**

Four hundred seventy-nine subjects without a history of diabetes underwent a 75 g 2 h Oral Glucose Tolerance Test (OGTT). New-onset diabetes (*n* = 101), pre-diabetes (*n* = 186), and normal glucose tolerance (*n* = 192) were screened. IR was defined by HOMA-IR > 2.69. Based on indices (HOMA-β, early-phase disposition index [DI_30_], (ΔIns30/ΔGlu30)/HOMA-IR and total-phase index [DI_120_]) that indicated different phases of insulin secretion, the subjects were divided into two groups, and the lower group was defined as having inadequate β cell compensation. Logistic regression models and accurate estimates of the areas under receiver operating characteristic curves (AUROC) were obtained.

**Results:**

In all of the subjects, TG/HDL, TC/HDL-C, LDL-C/HDL-C, and TG were significantly associated with IR. The AUROCs of TG/HDL-C and TG were 0.71 (95 % CI: 0.66–0.75) and 0.71 (95 % CI: 0.65–0.75), respectively. The optimal cut-offs of TG/HDL-C and TG for IR diagnosis were 1.11 and 1.33 mmol/L, respectively. The AUROCs of TC/HDL-C and LDL-C/HDL-C were 0.66 and 0.65, respectively, but they were not acceptable for IR diagnosis. TG/HDL-C,LDL-C/HDL-C and TG were significantly associated with HOMA-β, but AUROCs were less than 0.50; therefore, the lipid ratios could not be predictors of basal β cell dysfunction. None of the lipid ratios was associated with early-phase insulin secretion. Only TG/HDL-C and TG were significantly correlated with total-phase insulin secretion, but they also were not acceptable predictors of total-phase insulin secretion (0.60 < AUROC < 0.70).

**Conclusions:**

In a Chinese population with different levels of glucose tolerance, TG/HDL-C and TG could be the predictors of IR. The lipid ratios could not be reliable makers of β cell function in the population.

## Background

Insulin resistance (IR) and islet β cell dysfunction are two major risks in the pathogenesis of type 2 diabetes. The hyperinsulinemic/euglycemic clamp, which is regarded as the gold standard method to detect IR, is impractical for large clinical research studies [1]. IR is often accompanied by dyslipidemia. In pre-diabetic and diabetic patients, hyperglycemia was accompanied by dyslipidemia [2], and even in normoglycemic people [3]. Previous studies have reported that the triglyceride/high-density lipoprotein-cholesterol (TG/HDL-C) ratio was a surrogate marker of IR [4–6]; however, the relationship of TG/HDL-C with IR might vary by ethnicity [5, 7, 8]. The TG/HDL-C ratio could predict IR in Caucasians, while in African Americans, there remain conflicts in the association between TG/HDL-C and IR [9, 10], and several studies in Chinese subjects have suggested that TG/HDL-C could be a predictor of IR [11–14]. In contrast, the accurate assessment of islet β cell function is much more difficult than the assessment of insulin sensitivity [15]. Several studies have attempted to investigate the relationship between TG/HDL-C and β cell function. In normoglycemic African American women, TG/HDL-C was inversely associated with β cell function, suggesting that the TG/HDL-C ratio could be a simple tool for effectively identifying African Americans at risk for diabetes [16]. In our previous cohort study, a high baseline log (TG)/HDL-C ratio predicted rapid progression of islet β cell function [17]. However, the studies mentioned above focused on only one glucose tolerance status ranging from normal plasma glucose to diabetes. In fact, IR exists throughout all the glucose tolerance status, and whether TG/HDL-C could be a surrogate marker for IR and β cell function in populations with different levels of glucose tolerance remains unknown.

This study aimed to develop a simple predictive marker as a clinical tool for the evaluation of IR and β cell function in a Chinese population with different levels of glucose tolerance, ranging from normoglycemia to diabetes, to investigate whether lipid ratios could be potential clinical markers of IR and β cell function and to further explore the optimal cut-offs.

## Methods

### Study population

All subjects were recruited from a type 2 diabetes project in a Beijing suburb in China between March 2014 and January 2015. Four hundred eighty-nine subjects without a history of diabetes underwent a 75 g OGTT. The 75 g OGTT was conducted after an overnight fast (> 10 h). Blood samples were collected at 0 min, 30, 60 and 120 min following the OGTT. The glucose tolerance status of each subject was classified based on the 1999 criteria of the WHO: a normal glucose tolerance (NGT), indicated by fasting plasma glucose (FPG) < 6.1 mmol/L and 2 h postprandial glucose (2 h PG) < 7.8 mmol/L; pre-diabetes, indicated by impaired fasting glucose (IFT): 6.1 mmol/L ≤ FPG < 7.0 mmol/L and 2 h PG < 7.8 mmol/L; impaired glucose tolerance (IGT), indicated by FPG < 6.1 mmol/L and 7.8 ≤ 2 h PG <11.1 mmol/L; or IFT + IGT, with T2DM indicated by FPG ≥ 7.0 mmol/L or 2 h PG ≥ 11.1 mmol/L.

The subjects who have a current history of cigarette smoking and alcohol drinking were excluded, and subjects with serious diseases such as heart disease, stroke, kidney disease, liver disease, inflammatory disease were also excluded. Ten subjects who were on steroids or who were taking drugs interfering with lipid metabolism such as lipid-lowering agents, diuretics, β-blockers, fish oil were excluded. On the basis of the OGTT results, subjects with NGT (*n* = 101), pre-diabetes (*n* = 186), and diabetes (*n* = 192) were selected for this study. The study protocol was approved by the Ethics Committee of Peking Union Medical College Hospital. The subjects voluntarily signed informed consent forms.

### Clinical measurement

A standardized medical history and accurate physical examination were undertaken in all of the subjects before a 75 g OGTT was administered. Measurements of waist circumference (WC) (midway between the iliac crest and the costal margin) and hip circumference (HC) (at the level of the trochanters) were performed twice by the same observer, and the mean value was recorded. Weight and height were measured without shoes in light clothing, and body mass index (BMI) was calculated by dividing the body weight in kilograms by square of the height in meters. Blood pressure measurements were obtained twice with a standard mercury sphygmomanometer with the subjects at rest, and the mean value was calculated. Overweight and obesity were defined as 24 kg/m^2^ ≤ BMI < 28 kg/m^2^ and BMI ≥ 28 kg/m^2^, respectively [18].

### Biochemical measurements

Plasma glucose was measured by glucose oxidase assay. TC, TG, HDL-C, and LDL-C were determined using an automated analyzer. Serum insulin and C peptide were measured by chemiluminescent enzyme immunoassay. HbA1c analysis was performed by high-performance liquid chromatography (intra-assay CV < 3 %, inter-assay CV < 10 %).

### Assessment of IR

Homeostatic model assessment of insulin resistance was calculated as the following formula: (HOMA-IR). IR was defined as HOMA-IR >2.69, based on an epidemiology survey conducted in China [19].

### Assessment of β cell function

The homeostasis model assessment of insulin secretion (HOMA-β) was calculated as basal insulin release [20]. Early-phase insulin release was calculated as the total insulin area under the curve divided by the total glucose area under the curve during the first 30 min of the OGTT (InsAUC_30_/GluAUC_30_), which was shown to have a strong correlation with first-phase insulin secretion [21]. Insulin secretion relative to insulin sensitivity (ISI_M_: Matsuda insulin sensitivity index) was expressed as the disposition index (DI), calculated as: early-phase DI_30_ = [InsAUC_30_/GluACU_30_] × ISI_M_, (ΔIns30/ΔGlu30)/HOMA-IR and total-phase DI_120_ = [InsAUC_120_/GluACU_120_] × ISI_M_. Another formula for assessing early-phase insulin release was: (ΔIns_30_/ΔGlu_30_)/HOMA-IR.

There were no prior data on the alternate cut-offs for inadequate β cell compensation in Chinese. In the present study, subjects with pre-diabetes/diabetes accounted for 59.9 % of the sample; therefore, based on the indices above, the subjects were divided into two groups. We defined the lower group as having inadequate β cell compensation.

### Statistical analysis

All of the statistical analyses were performed using SPSS software, version 17.0 (Chicago, IL, USA). The data are presented as the means ± SDs. Parameters not normally distributed were transformed. Categorical data were analyzed using the *χ*^2^ test. The significance of the mean differences was tested by the *t* test and ANOVA (followed by Bonferroni’s post hoc pairwise comparisons).

All P- values were two-sided, and P <0.05 was considered statistically significant.

To explore the associations among the lipid ratios (TG/HDL-C, TC/HDL-C, LDL-C/HDL-C), TG and IR, and β cell function, logistic regression models were used, and odds ratios (ORs) and 95 % confidence interval (CIs) were calculated. First, single factor analysis was conducted, and lipid ratios and TG, sex, age, BMI, WC, and HC were used as independent variables; then, in the multivariable analysis model, the confounding factors that were significantly associated with IR and β cell function in single factor analysis were added.

The lipid ratios that were significantly associated with IR or β cell function in the multivariable analysis model were used to estimate the area under the receiver operating characteristic (AUROC) curve for analysis. The AUROCs were also adjusted for the covariates used in the logistic models. Based on the AUROCs, the diagnostic value of the lipid ratios and TG were assessed: an AUROC ≤ 0.5 was considered no discrimination, an AUROC between 0.7 and 0.8 was considered acceptable, an AUROC between 0.8 and 0.9 was considered excellent, and an AUROC > 0.9 was considered outstanding [22]. Youden’s index was calculated as (specificity + sensitivity − 1) and was used to select the optimal cut-offs for each lipid ratio and TG.

## Results

1. Clinical characteristics of subgroups divided by plasma glucose profiles and insulin resistance

According to the plasma glucose levels, the subjects were divided into three groups: normal glucose tolerance (NGT), pre-diabetes and diabetes. The characteristics among the three groups are presented in Table 1. The pre-diabetes and diabetes subjects had older ages, larger BMIs, waist circumferences, hip circumferences, proportions with overweight/obesity and higher SBP than the normal glycemic tolerance subjects. In both pre-diabetes and diabetes subjects, insulin (30 min), C peptide (30 min), HOMA-β, DI_30_, DI_120_, and (ΔIns_30_/ΔGlu_30_)/HOMA-IR were significantly lower than those in the normal glycemic subjects. The diabetes subjects had higher lipid profiles (TG, TG/HDL-C, TC/HDL-C, LDL-C/HDL-C) and HOMA-IR than the normal glycemic and pre-diabetes subjects.

**Table 1:** Characteristics in different glucose tolerance status

Characteristic	NGT	Pre-diabetes	Diabetes	*P*-value
	*N* = 192	*N* = 186	*N* = 101	
Female (%)	135 (70.31)	115 (61.83)	60 (59.41)	0.000*
Age, years	48.77 ± 11.55	55.36 ± 10.22	54.54 ± 9.89	0.000*
BMI, kg/m^2^	24.98 ± 3.46	26.74 ± 3.74	26.86 ± 4.15	0.000*
Overweight/obesity (%)	114 (59.38)	136 (73.12)	80 (79.21)	0.000*
Waist circumference, cm	84.64 ± 9.75	88.48 ± 9.19	88.31 ± 9.54	0.000*
Hip circumference, cm	90.17 ± 9.88	93.52 ± 8.88	93.13 ± 12.26	0.003*
Systolic BP, mm Hg	123.59 ± 19.08	129.12 ± 15.92	131.45 ± 20.89	0.001*
Diastolic BP, mm Hg	75.54 ± 9.93	76.50 ± 10.02	77.06 ± 10.76	0.429
HbA1c	5.26 ± 0.29	5.71 ± 0.33	6.87 ± 1.42	0.000*
Fasting plasma glucose (PG), mmol/L	5.45 ± 0.35	6.09 ± 0.48	8.48 ± 2.77	0.000*
PG 30’, mmol/L	8.96 ± 1.93	10.71 ± 2.01	14.78 ± 3.97	0.000*
PG 60’, mmol/L	7.57 ± 1.98	10.09 ± 2.82	16.73 ± 4.92	0.000*
PG 120’, mmol/L	5.86 ± 1.18	7.61 ± 1.76	14.92 ± 5.79	0.000*
Ln (Ins 0’, mU/L)	2.19 ± 0.49	2.31 ± 0.51	2.40 ± 0.65	0.004*
Ln (Ins 30’, mU/L)	4.28 ± 0.67	4.19 ± 0.65	3.65 ± 0.83	0.000*
Ln (Ins 60’, mU/L)	4.09 ± 0.66	4.29 ± 0.69	4.05 ± 0.90	0.008*
Ln (Ins 120’, mU/L)	3.44 ± 0.72	3.87 ± 0.78	3.96 ± 0.96	0.000*
Ln (C peptide 0’, ng/mL)	0.18 ± 0.36	0.36 ± 0.39	0.40 ± 0.48	0.000*
Ln (C peptide 30’, ng/mL)	1.63 ± 0.42	1.57 ± 0.42	1.15 ± 0.59	0.000*
Ln (C peptide 60’, ng/mL)	1.74 ± 0.44	1.84 ± 0.90	1.58 ± 0.61	0.000*
Ln (C peptide 120’, ng/mL)	1.47 ± 0.42	1.76 ± 0.44	1.71 ± 0.63	0.000*
InsAUC_30_/GluAUC_30_ (mU/mmol)	6.84 ± 2.36	5.72 ± 2.41	3.11 ± 1.72	0.000*
InsAUC_120_/GluAUC_120_ (mU/mmol)	8.39 ± 2.77	9.21 ± 2.02	5.04 ± 2.38	0.000*
Ln HOMA-IR	0.79 ± 0.46	1.02 ± 0.50	1.38 ± 0.68	0.000*
Sqrt HOMA-β	9.99 ± 2.55	9.20 ± 2.43	7.68 ± 2.86	0.000*
Sqrt DI_30_	24.91 ± 4.90	19.58 ± 4.64	11.97 ± 4.18	0.000*
Sqrt DI_120_	27.91 ± 3.84	23.39 ± 6.78	15.06 ± 6.01	0.000*
Ln [(ΔIns_30_/ΔGlu_30_)/HOMA-IR]	2.32 ± 0.78	1.76 ± 0.71	0.85 ± 0.59	0.000*
Total cholesterol (TC), mmol/L	5.26 ± 1.04	5.64 ± 1.01	5.61 ± 0.97	0.001*
Log (triglyceride [TG]), mmol/L	0.09 ± 0.27	0.20 ± 0.24	0.26 ± 0.26	0.000*
Log (HDL-C, mmol/L)	0.11 ± 0.10	0.10 ± 0.10	0.08 ± 0.09	0.013*
LDL-C, mmol/L	2.67 ± 0.74	2.96 ± 0.69	2.91 ± 0.66	0.000*
Log (TG/HDL-C)	−0.02 ± 0.32	0.10 ± 0.29	0.18 ± 0.30	0.000*
TC/HDL-C	4.08 ± 1.02	4.50 ± 0.91	4.66 ± 0.85	0.000*
LDL-C/HDL-C	2.09 ± 0.71	2.37 ± 0.64	2.43 ± 0.60	0.000*

* Significant results: *p* < 0.05

According to IR, the subjects were divided into an insulin resistance group (HOMA-IR >2.69) and insulin sensitivity group (HOMA-IR ≤2.69). The characteristics are presented in Table 2. There was no significant difference in age, sex, blood pressure, or total cholesterol between the groups. The proportions with diabetes and overweight/obesity, the lipid profiles (TG, LDL-C, TG/HDL-C, TC/HDL-C, LDL-C/HDL-C), fasting and postprandial plasma glucose, insulin, and C peptide were much higher in the HOMA-IR >2.69 group. HOMA-β, which reflects the basal insulin secretion, was significantly higher in the elevated HOMA-IR group, while DI_30_ and (ΔIns_30_/ΔGlu_30_)/HOMA-IR, which indicate early-phase insulin secretion, and DI_120_, which reflects total-phase insulin secretion, were significantly lower in the elevated HOMA-IR group.

**Table 2:** Characteristics of insulin sensitivity vs. insulin resistance

Characteristic	Insulin sensitivity	Insulin resistance	*P*-value
	HOMA-IR ≤ 2.69	HOMA-IR > 2.69	
	*N* = 241	*N* = 238	
Diabetes (%)	29 (12.03)	72 (30.25)	0.000*
Pre-diabetes (%)	87 (36.10)	99 (41.60)	0.217
NGT (%)	125 (51.87)	67 (28.15)	0.000
Female (%)	153 (63.49)	158 (66.39)	0.468
Age, years	53.06 ± 10.99	51.92 ± 11.25	0.267
BMI, kg/m^2^	24.39 ± 3.13	27.76 ± 3.68	0.000*
Overweight/obesity (%)	124 (51.45)	206 (86.55)	0.000*
Waist circumference, cm	83.96 ± 9.38	89.88 ± 8.97	0.000*
Hip circumference, cm	89.30 ± 9.38	89.88 ± 8.97	0.000*
Systolic BP, mm Hg	127.22 ± 19.17	127.65 ± 18.03	0.804
Diastolic BP, mm Hg	75.83 ± 10.19	76.83 ± 10.11	0.391
HbA1c%	5.58 ± 0.76	5.97 ± 1.04	0.000*
Fasting plasma glucose, mmol/L	5.90 ± 1.40	6.78 ± 1.95	0.000*
Postprandial glucose (PG 30’), mmol/L	10.06 ± 2.97	11.67 ± 3.47	0.000*
PG 60’, mmol/L	9.42 ± 3.97	11.54 ± 4.99	0.000*
PG 120’, mmol/L	7.44 ± 3.56	9.44 ± 5.15	0.000*
Ln (Ins 0’, mU/L)	1.88 ± 0.33	2.69 ± 0.36	0.000*
Ln (Ins 30’, mU/L)	3.83 ± 0.67	4.41 ± 0.69	0.000*
Ln (Ins 60’, mU/L)	3.84 ± 0.62	4.48 ± 0.46	0.000*
Ln (Ins 120’, mU/L)	3.37 ± 0.73	4.07 ± 0.70	0.000*
Ln (C peptide 0’, ng/mL)	0.02 ± 0.28	0.58 ± 0.31	0.000*
Ln (C peptide 30’, ng/mL)	1.35 ± 0.44	1.67 ± 0.49	0.000*
Ln (C peptide 60’, ng/mL)	1.58 ± 0.42	1.91 ± 0.46	0.000*
Ln (C peptide 120’, ng/mL)	1.46 ± 0.46	1.82 ± 0.46	0.000*
InsAUC_30_/GluAUC_30_ (mU/mmol)	4.18 ± 2.80	7.08 ± 5.02	0.000*
InsAUC_120_/GluAUC_120_ (mU/mmol)	5.60 ± 3.09	10.44 ± 4.97	0.000*
Sqrt HOMA-β	7.94 ± 1.88	10.45 ± 2.83	0.000*
Sqrt DI_30_	21.79 ± 6.27	18.47 ± 6.75	0.000*
Sqrt DI_120_	25.33 ± 5.97	21.59 ± 8.13	0.000*
Ln [(ΔIns_30_/ΔGlu_30_)/HOMA-IR]	2.01 ± 0.91	1.58 ± 0.84	0.000*
Total cholesterol (TC), mmol/L	5.41 ± 0.95	5.56 ± 1.09	0.113
Log (triglyceride [TG]) mmol/L)	0.08 ± 0.22	0.26 ± 0.27	0.000*
Log (HDL-C, mmol/L)	0.12 ± 0.10	0.08 ± 0.08	0.000*
LDL-C, mmol/L	2.73 ± 0.68	2.94 ± 0.73	0.001*
Log (TG/HDL-C)	−0.04 ± 0.28	0.18 ± 0.30	0.000*
TC/HDL-C	4.12 ± 0.94	4.62 ± 0.94	0.000*
LDL-C/HDL-C	2.10 ± 0.67	2.45 ± 0.64	0.000*

* Significant results: *p* < 0.05

2. Association of lipid ratios with IR in the population with different levels of glucose tolerance

In the whole population with continuous glucose tolerance, TG/HDL-C, TC/HDL-C, LDL-C/HDL-C, TG, and HDL-C were significantly associated with IR. Single factor analysis showed that sex, BMI, waist circumference, hip circumference, and plasma glucose were associated with IR (Table 3), and these confounding factors were included in the binary multivariable logistic regression model analysis, in which TG/HDL-C, TC/HDL-C, LDL-C/HDL-C, and TG were significantly associated with IR, and the association was independent of these confounding factors (Table 4). Therefore, the next step was to explore whether TG/HDL-C, TC/HDL-C, LDL-C/HDL-C, and TG could be good predictors of IR.

**Table 3:** Single factor analysis: association of lipid ratios, TG, clinical features and insulin resistance

	Total population		Female (*n* = 310)		Male (*n* = 169)	
	OR (95 % CI)	*P*-value	OR (95 % CI)	*P*-value	OR (95 % CI)	*P*-value
Log (TG/HDL-C)	14.38 (7.00–29.51)	0.000*	17.68 (6.83–45.76)	0.000*	19.17 (5.72–64.28)	0.000*
TC/HDL-C	1.78 (1.45–2.20)	0.000*	2.03 (1.54–2.67)	0.000*	1.69 (1.20–2.37)	0.003*
LDL-C/HDL-C	2.26 (1.68–3.03)	0.000*	2.74 (1.86–4.03)	0.000*	2.00 (1.23–3.25)	0.005*
Log TG	22.18 (9.30–52.93)	0.000*	24.77 (7.80–76.72)	0.000*	27.27 (6.50–114.32)	0.000*
Log HDL-C	0.01 (0.001–0.06)	0.000*	0.003 (0.00–0.05)	0.000*	0.01 (0.00–0.22)	0.005*
Sex	0.68 (0.46–0.99)	0.042*	——	——	——	——
Age, years	0.99 (0.98–1.01)	0.267	1.02 (0.99–1.04)	0.094	0.94 (0.91–0.97)	0.000*
BMI, kg/m^2^	1.36 (1.27–1.45)	0.000*	1.29 (1.19–1.39)	0.000*	1.59 (1.36–1.85)	0.000*
Waist circumference, cm	1.07 (1.05–1.10)	0.000*	1.08 (1.06–1.11)	0.000*	1.09 (1.05–1.13)	0.000*
Hip circumference, cm	1.06 (1.04–1.09)	0.000*	1.08 (1.05–1.11)	0.000*	1.06 (1.02–1.10)	0.001*
Systolic BP, mm Hg	1.00 (0.99–1.01)	0.790	0.99 (0.99–1.01)	0.705	1.01 (0.99–1.03)	0.273
Diastolic BP, mm Hg	1.00 (0.99–1.03)	0.383	1.01 (0.99–1.03)	0.389	1.01 (0.98–1.04)	0.658
Diabetes	4.63 (2.75–7.82)	0.000*	4.25 (2.18–8.30)	0.000*	6.62 (2.71–16.15)	0.000*
Pre-diabetes	2.15 (1.42–3.25)	0.000*	2.24 (1.35–3.72)	0.002*	2.44 (1.14–5.25)	0.022*

* Significant results: p < 0.05

**Table 4:** Multivariable binary logistic regression analysis: the associations of lipid ratios, TG and insulin resistance

	Total population		Female		Male	
	OR (95 % CI)	*P*-value	OR (95 % CI)	*P*-value	OR (95 % CI)	*P*-value
Log (TG/HDL-C)	6.27 (2.86–13.76)	0.000*	5.91 (2.12–16.46)	0.001*	4.58 (1.18–17.85)	0.028*
TC/HDL-C	1.39 (1.09–1.75)	0.006*	1.39 (1.03–1.88)	0.031*	1.36 (0.89–2.07)	0.117
LDL-C/HDL-C	1.63 (1.17–2.27)	0.004*	1.63 (1.06–2.51)	0.025*	1.57 (0.85–2.90)	0.147
Log TG	7.79 (3.06–19.83)	0.000*	6.94 (2.01–23.98)	0.002*	5.43 (1.14–25.97)	0.034*
Log HDL-C	0.31 (0.13–0.72)	0.310	0.03 (0.00–0.61)	0.023*	0.12 (0.00–11.36)	0.360

* Significant results: *p* < 0.05

In women, TG/HDL-C, TC/HDL-C, LDL-C/HDL-C, TG, and HDL-C were significantly associated with IR, and in men, TG/HDL-C and TG were associated with IR, independent of age, BMI, waist circumference, hip circumference, and plasma glucose profile (Table 4).

3. Comparison of area under ROCs and optimal cut-offs for predictors of IR in a population with different levels of glucose tolerance

**Fig. 1:**
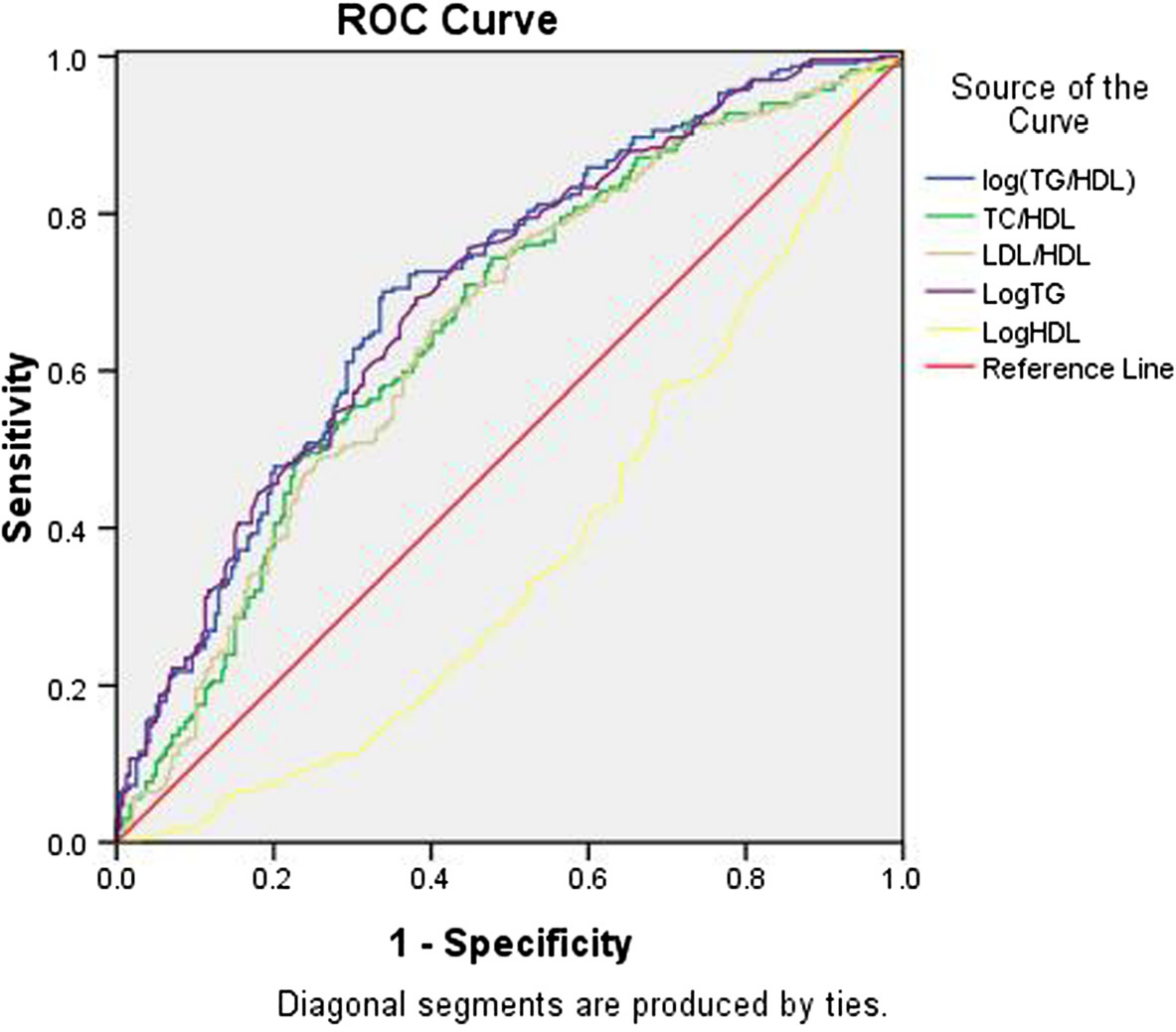
Area under the receiver operating characteristics curves (AUROCs) of the lipid markers for insulin resistance

In the study population, the AUROCs for TG/HDL-C and TG were 0.71 (95 % CI: 0.66–0.75) and 0.71 (95 % CI: 0.65–0.75), respectively (Table 5, Fig. 1), and the optimal cut-offs for TG/HDL-C and TG were 1.11 (sensitivity: 70.1 %, specificity: 66.1 %) and 1.33 (sensitivity: 69.2 %, specificity: 61.9 %). TG/HDL-C and TG (the AUROC >0.70) were acceptable predictors of IR defined by HOMA-IR. The AUROCs for TC/HDL-C (0.60 < AUROC < 0.70), LDL-C/HDL-C (0.60 < AUROC < 0.70), and HDL-C (AUROC < 0.50) were lower (Table 5, Fig. 1).

**Table 5:** Area under the receiver operating characteristics curves (ROCs) of the lipid markers for insulin resistance in the whole population

	AUROC (95 % CI)	*P*-value
Log (TG/HDL-C)	0.71 (0.66–0.75)	0.000*
TC/HDL-C	0.66 (0.61–0.71)	0.000*
LDL-C/HDL-C	0.65 (0.60–0.70)	0.000*
Log TG	0.71 (0.65–0.75)	0.000*
Log HDL-C	0.37 (0.32–0.42)	0.000*

* Significant results: *p* < 0.05

In women, the AUROC for TG/HDL-C was 0.70 (95 % CI: 0.65–0.76), and the AUROCs for TC/HDL-C, LDL-C/HDL-C, and TG were relatively smaller (0.60 < AUROC < 0.70). In men, the AUROCs for TG/HDL-C and TG were small (AUROC < 0.50).

4. Association of lipid ratios with islet β cell function in a population with different levels of glucose tolerance

In the whole population, TG/HDL, LDL-C/HDL-C and TG were significantly associated with HOMA-β (Table 6). In the binary multivariable logistic regression model analysis, TG/HDL-C, LDL-C/HDL-C and TG were negatively associated with HOMA-β, independent of age, sex, BMI, glucose profile and IR (Table 7). The associations among TG/HDL, LDL-C/HDL-C, TG and HOMA-β still existed when the subjects were grouped by sex, both in female and male subjects. The next step was to explore whether TG/HDL-C and TG could be acceptable predictors of fasting β cell dysfunction.

**Table 6:** Single factor analysis: association of lipid ratios, TG, clinical features and insulin secretion in different phases

	HOMA-β		DI_30_		(ΔIns30/Glu30)/HOMA-IR		DI_120_	
	OR (95 % CI)	*P*-value	OR (95 % CI)	*P*-value	OR (95 % CI)	*P*-value	OR (95 % CI)	*P*-value
Log (TG/HDL-C)	0.46 (0.25–0.83)	0.010*	4.20 (2.22–7.94)	0.000*	2.66 (1.45–4.88)	0.002*	5.67 (2.94–10.92)	0.000*
TC/HDL-C	0.89 (0.74–1.07)	0.226	1.54 (1.26–1.89)	0.000*	1.36 (1.21–1.66)	0.002*	1.61 (1.31–1.97)	0.000*
LDL-C/HDL-C	0.76 (0.58–1.00)	0.050	1.72 (1.30–2.29)	0.000*	1.47 (1.11–1.94)	0.007*	1.80 (1.35–2.40)	0.000*
Log TG	0.41 (0.20–0.83)	0.013*	5.80 (2.68–12.54)	0.000*	3.24 (1.57–6.72)	0.002*	8.01 (3.62–17.72)	0.000*
Log HDL-C	3.95 (0.57–27.31)	0.164	0.15 (0.02–1.09)	0.061	0.26 (0.04–1.82)	0.175	0.07 (0.01–0.52)	0.010*
Sex	2.48 (1.69–3.66)	0.000*	1.30 (0.90–1.90)	0.176	1.39 (0.95–2.04)	0.089	1.57 (1.07–2.30)	0.021*
Age, years	1.05 (1.03–1.07)	0.000*	1.04 (1.02–1.06)	0.000*	1.04 (1.02–1.06)	0.000*	1.05 (1.03–1.07)	0.000*
BMI, kg/m^2^	0.91 (0.87–0.96)	0.000*	1.10 (1.04–1.15)	0.000*	1.07 (1.02–1.13)	0.006*	1.11 (1.06–1.18)	0.000*
Waist circumference, cm	0.99 (0.97–1.01)	0.270	1.04 (1.02–1.06)	0.000*	1.03 (1.01–1.05)	0.007*	1.03 (1.01–1.06)	0.001*
Hip circumference, cm	0.99 (0.97–1.01)	0.250	1.03 (1.01–1.05)	0.003*	1.02 (1.00–1.04)	0.052	0.01 (1.01–1.04)	0.012*
Systolic BP, mm Hg	1.01 (0.10–1.02)	0.093	1.02 (1.01–1.03)	0.004*	1.01 (1.00–1.02)	0.032*	1.01 (1.00–1.02)	0.009*
Diastolic BP, mm Hg	1.01 (0.99–1.02)	0.513	1.01 (0.99–1.03)	0.414	1.01 (0.99–1.03)	0.281	1.01 (0.99–1.03)	0.276
Diabetes	4.22 (2.51–7.09)	0.000*	129.66 (44.21–380.26)	0.000*	79.69 (30.21–210.23)	0.000*	141.19 (51.76–385.18)	0.000*
Pre-diabetes	2.07 (1.37–3.13)	0.001*	8.80 (5.35–14.46)	0.000*	5.77 (3.62–9.21)	0.000*	14.68 (8.55–25.23)	0.000*
HOMA-IR	0.68 (0.60–0.76)	0.000*	2.98 (2.08–4.28)	0.000*	2.46 (1.70–3.57)	0.000*	3.07 (2.10–4.48)	0.000*

* Significant results: *p* < 0.05

**Table 7:** Multivariable binary logistic regression analysis: the association of lipid ratios, TG and insulin secretion in different phases

	HOMA-β		DI_30_		(ΔIns30/Glu30)/HOMA-IR		DI_120_	
	OR (95 % CI)	*P*-value	OR (95 % CI)	*P*-value	OR (95 % CI)	*P*-value	OR (95 % CI)	*P*-value
Log (TG/HDL-C)	0.27 (0.12–0.57)	0.001*	1.79 (0.79–4.04)	0.164	0.90 (0.40–2.04)	0.806	2.46 (1.06–5.74)	0.036*
TC/HDL-C	0.74 (0.59–0.94)	0.743	1.12 (0.86–1.46)	0.392	0.97 (0.75–1.25)	0.792	1.09 (0.82–1.46)	0.548
LDL-C/HDL-C	0.58 (0.41–0.81)	0.001*	1.15 (0.79–1.67)	0.470	0.94 (0.66–1.36)	0.751	1.07 (0.71–1.61)	0.744
Log TG	0.22 (0.90–0.54)	0.001*	2.15 (0.84–5.49)	0.109	0.97 (0.97–0.38)	0.949	2.84 (1.04–7.72)	0.041*
Log HDL-C	6.78 (0.71–64.58)	0.096	1.17 (0.08–16.24)	0.908	2.60 (0.20–33.19)	0.462	0.81 (0.04–14.83)	0.886

* Significant results: *p* < 0.05

In all of the subjects, TG/HDL-C, TC/HDL-C, LDL/HDL-C, and TG were significantly associated with the indices of early-phase insulin secretion function (DI_30_, [ΔIns_30_/ΔGlu_30_]/HOMA-IR) (Table 6). Considering the confounding factors, such as age, BMI, waist circumferences, and hip circumference, in the multivariable logistic regression model, the lipid ratios and TG were not significantly associated with the indices of early-phase insulin secretion function (Table 7).

TG/HDL-C and TG were significantly positively associated with DI_120_, and were independent of age, BMI, waist circumference, hip circumference, glucose profile and IR. TC/HDL-C and LDL/HDL-C was not associated with DI_120_ (Table 7). The associations among TG/HDL, TG and DI_120_ still existed when the subjects were grouped by sex, both in female and male subjects. The next step was to explore whether TG/HDL-C and TG could be good predictors of total phase β cell dysfunction.

5. Comparison of area under ROCs and optimal cut-offs for predictors of islet β cell dysfunction in a population with continuous glucose tolerance status

The AUROCs of TG/HDL-C, LDL-C/HDL-C and TG for basal β cell secretion were small (AUROC < 0.50); therefore, lipid ratios could not be predictors of basal β cell dysfunction. TG/HDL-C and TG also could not be acceptable predictors of total phase insulin secretion (0.60 < AUROC < 0.70). When the population was divided by sex, both in women and in men, the AUROCs for TG/HDL-C, LDL-C/HDL-C, and TG were small (AUROC < 0.50).

## Discussion

The study showed that insulin resistance, the lipid ratios (TG/HDL-C, TC/HDL-C, LDL-C/HDL-C) and TG increased, while basal, early-phase, and total phase insulin secretion decreased in the population with different glucose tolerance status from normal plasma glucose to diabetes. TG/HDL-C and TG could be serum predictors of insulin resistance in the whole population (TG/HDL-C: AUROC: 0.71, 95 % CI: 0.66–0.75; TG: AUROC: 0.71, 95 % CI: 0.65–0.75); the optimal cut-offs for TG/HDL and TG were 1.11 and 1.33 mmol/L, respectively. Many studies have shown that increasing TG and decreasing HDL-C could cause insulin resistance. When circulating TG was at high levels, heparin activated lipoprotein lipase to increase intravascular lipolysis of TG, thus increasing the risk of tissue exposure to free fatty acids (FFAs). High FFAs could result in insulin resistance via oxidative stress pathways [10, 23]. Previous studies have suggested that the prediction of lipid ratios for IR was influenced by confounding factors, such as sex, age, and BMI. In our multivariable analysis, considering the confounding factors, such as sex, age, BMI, and plasma glucose profiles, the associations among TG/HDL-C, TG and IR remained. In the present study, the plasma glucose spectrum of the subjects was closer to that in the real population, ranging from normal glucose tolerance to diabetes. The study was the first in a population with different levels of glucose tolerance demonstrating that TG/HDL-C and TG could be predictors of IR, the results were in agreement with previous studies based on normal plasma glucose Chinese populations [11–14].

The present study explored the associations between lipid ratios and insulin secretion function in different phases, TG/HDL-C and TG were significantly negatively associated with basal insulin secretion and positively associated with total-phase insulin secretion. The lipid ratio could not be a reliable marker of β cell function in the population. Lipid ratios were not correlated with early-phase insulin secretion, the result was not in accordance with study in normoglycemic and pre-diabetic Japanese subjects, which showed that TG/HDL-C and TG were negatively associated with early-phase insulin secretion [2]. Considering that our subjects had new onset pre-diabetes/diabetes, the total insulin secretion increased under the conditions of hyperglycemia. Among these new onset patients, insulin secretion was affected by insulin sensitivity. In the present study, although the indices (DI_30_, [ΔIns_30_/ΔGlu_30_]/HOMA-IR, DI_120_) had already been adjusted according to insulin sensitivity, the effect of insulin sensitivity on insulin secretion could not be entirely ignored. Although in our former cohort study of patients with type 2 diabetes, high baseline log (TG)/HDL-C ratio could be a predictor of decreased β cell function [17], in the present study the AUROCs of TG/HDL-C and TG for β cell function were relatively low, the predictive accuracy of TG/HDL-C and TG for β cell function was limited. The role of lipid ratios for predicting β cell function is still controversial in population in different ethics and in different glucose tolerances, a recent study in normoglycemic African American women showed that TG/HDL-C could predict β cell function [16]. A cross-sectional study in normoglycemic Chinese subjects suggested that there was no relationship between TG/HDL-C and β cell function [24].

The study had some limitations. This study was a cross-sectional study to investigate the relationship between lipid and insulin resistance, β cell function, however, longitudinal study was more convincing than cross-sectional study. We cannot assess the lifestyle like exercise and the status of menopause in women exactly, which may be confounders in the study. In future, further study should be conduct to confirm the conclusion.

## Conclusion

The present study was the first in a population with different levels of glucose tolerance demonstrating that TG/HDL-C and TG could be predictors of IR. TG/HDL-C and TG were significantly negatively associated with basal insulin secretion and positively associated with total-phase insulin secretion, however, the lipid ratio could not be a reliable marker of β cell function in the population.
